# Effect of integrating traditional care with modern healthcare to improve tuberculosis control programs in Ethiopia: a protocol for a cluster-randomized controlled trial

**DOI:** 10.1186/s13063-023-07559-8

**Published:** 2023-09-11

**Authors:** Desalegne Amare, Fentie Ambaw, Kefyalew Addis Alene

**Affiliations:** 1 School of Health Sciences, Bahir Dar University, Bahir Dar, Ethiopia; 2 School of Public Health, Bahir Dar University, Bahir Dar, Ethiopia; 3 School of Population Health, Faculty of Health Sciences, Curtin University, Perth, Australia; 4 Geospatial and Tuberculosis Research Team, Telethon Kids Institute, Nedlands, Australia

**Keywords:** Tuberculosis, Traditional health care, Service integration, Cluster-randomized controlled trial, Ethiopia

## Abstract

**Background:**

Tuberculosis (TB) remains a major cause of morbidity and mortality in the world, despite being a preventable and curable disease. The World Health Organization (WHO) End-TB Strategy, aligned with the Sustainable Development Goals (SDGs), sets a target of reducing the TB mortality rate by 95%, TB incidence rate by 90%, and catastrophic costs due to TB by 2035, compared with a 2015 level. To achieve these ambitious targets, several interventions have been implemented in the last few years, resulting in major progress toward reducing the burden of TB. However, over one-third of the global TB cases remained undetected and never received treatment. Most of those undetected cases were found in low- and middle-income countries such as Ethiopia. Though several interventions were implemented to increase TB case detection and mitigate catastrophic costs associated with TB, sustaining these interventions in resource-constrained settings remains challenging. Consequently, an alternative method is needed to increase TB case detection while decreasing diagnosis delays and catastrophic costs. Therefore, this study aimed to integrate traditional TB care into modern TB care to improve TB control programs, including early TB case detection, and reduce catastrophic costs in high TB burden settings such as Ethiopia.

**Methods:**

A cluster randomized controlled trial will be conducted in northwest Ethiopia to determine the effectiveness of integrating traditional care with modern TB care. The intervention will be conducted in randomly selected districts in the South Gondar Zone. The control group will be an equal number of districts with usual care. The intervention comprised three key components, which include referral linkage from traditional to modern health care; training of health professionals and traditional care providers in three different rounds to increase their knowledge, attitude, and skills toward the referral systems; and TB screening at traditional health care sites. The primary outcomes of interest will be an increase in case detection rate, and the secondary outcomes of interest will be decreased diagnosis delays and catastrophic costs for TB patients. Data will be collected in both the intervention and control groups on the main outcome of interest and a wide range of independent variables. Generalized linear mixed models will be used to compare the outcome of interest between the trial arms, with adjustment for baseline differences.

**Discussion:**

This cluster-randomized controlled trial study will assess the effectiveness of a strategy that integrates traditional healthcare into the modern healthcare system for the control and prevention of TB in northwest Ethiopia, where nearly 90% of the population seeks care from traditional care systems. This trial will provide information on the effectiveness of traditional and modern healthcare integration to improve TB case detection, early diagnosis, and treatment, as well as reduce the catastrophic costs of TB.

**Trial registration:**

ClinicalTrials.gov NCT05236452. Registered on July 22, 2022.

**Supplementary Information:**

The online version contains supplementary material available at 10.1186/s13063-023-07559-8.

## Background

Most people with TB can fully recover from the disease with early diagnosis and treatment. However, it continues to be a leading cause of death worldwide, killing over one million people each year [1–3]. In 2020, an estimated 10 million people developed TB, and 1.4 million died as a result of TB. Of these, more than 87% of the global TB cases were reported in 30 TB high-burden countries [4]. Africa is one of the most affected regions, accounting for one-fourth (25%) of the global TB burden [5]. In Ethiopia, the incidence of TB was 140 cases per 100,000 people in 2020 [6].

The WHO has ambitious targets of a 95% reduction in TB deaths, a 90% reduction in TB incidence, and no catastrophic costs due to TB by 2035 [7]. However, according to a WHO report, approximately 4.1 million people with TB will not have been diagnosed or reported to national authorities by 2021 [8]. Most of these undetected cases were found in low- and middle-income countries or countries with weak healthcare systems. Under-detection of TB cases is also the most common problem in Ethiopia [9–11] with a high magnitude of undiagnosed smear-positive pulmonary TB (PTB) cases found in the community [12]. According to the 2021 national TB prevention and control guideline, one-third of patients with active TB remain undiagnosed and untreated in Ethiopia, and 39% of cases were not reported or not diagnosed in the Amhara region [13].

Although WHO planned to reach zero catastrophic costs, TB still causes financial hardships for the affected families [14]. Although TB medications and laboratory tests are offered free of charge in developing countries, including Ethiopia, the direct and indirect costs associated with TB are still very high [15]. TB patients face a high cost due to charges for health services in private clinics, costs for transport, accommodation, nutrition, and lost income due to the inability to work [16, 17].

Delays in the diagnosis and treatment of TB lead to slow progression of the disease, poor treatment outcomes, and an increased risk of TB transmission [18]. Delayed TB diagnosis can result in physical, social, and mental health problems such as depression, anxiety, and psychosis [19]. It can also affect social life and impose an economic burden on TB-affected families [20–22]. TB diagnosis delay in Ethiopia is unacceptable, with a previous study showing that 91% of TB patients exceeded 31 days in total delay [23]. Delays in TB diagnosis were also high in the Amhara region, with three-fifths of patients having delays in TB diagnosis and treatment [24]. The lack of appropriate intervention was the main contributor to the delay in TB diagnosis [25].

Since more people first visit traditional care, they delay visiting the modern healthcare system. Studies show that the first visit to traditional healers can lead to lower case detection, longer diagnostic delays, and higher costs. A study conducted by the WHO showed that 70–80% of the population in developing countries uses traditional medicine [26]. Traditional medicine encompasses knowledge and practices that are inherent to various cultures, whether or not they can be rationalized, and that are used to maintain health as well as to prevent, diagnose, treat, and improve physical and mental illness [27].

**Fig. 1:**
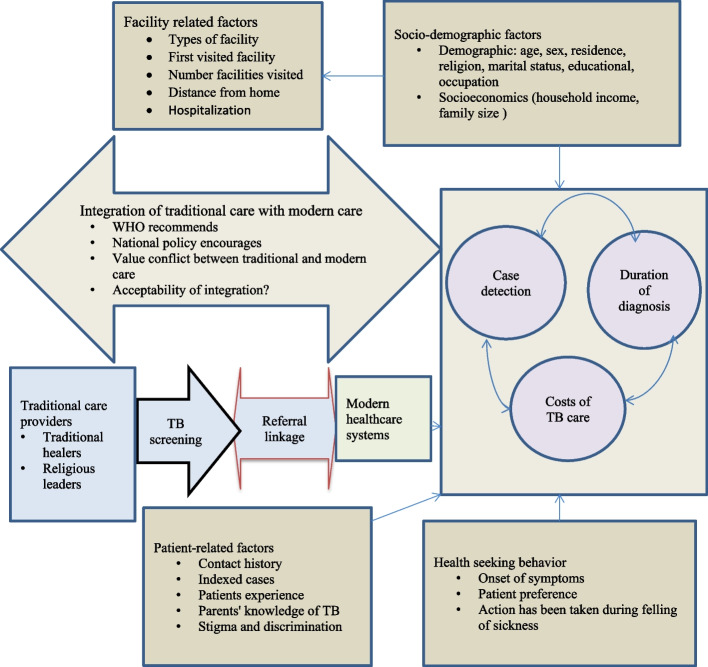
Conceptual framework showing the integrated systems between traditional care and modern health care system to to increase case detection rate, reduce the diagnosis and treantmet delays and catastrophic costs of TB in northwest Ethiopia

A recent study conducted in Ethiopia showed that more than 70% of the participants sought treatment from traditional or religious healers for a minimum of 6 months before eventually seeking care from the modern healthcare system [28]. The utilization of traditional or religious healers for an extended period before seeking modern healthcare services may have played a significant role in causing delays in diagnosing TB. Thus, integrating traditional TB care with the modern healthcare system may be important to increase case detection, reduce diagnosis delays, and reduce catastrophic costs. Integrating traditional TB care with modern care can also bridge the gap between conventional practices and contemporary medical advancements, fostering a holistic and patient-centric approach to TB management. It may also help to create a more comprehensive, accessible, and patient-friendly TB care system. Figure 1 shows the pathway and contributing factors for the low detection rate, diagnosis delay, and catastrophic cost. While the literature lacks a precise definition of “integrating traditional TB care with modern care,” for the purpose of this study, it is defined as the collaborative effort between traditional care practitioners and modern care practitioners through referral linkage and training.

The following are the specific objectives:

- To determine the effect of traditional and modern care in reducing TB diagnosis delay
- To estimate the effect of integrating traditional care with modern care on the cost of TB care among tuberculosis patients
- To determine the effect of integrating traditional care into modern care in increasing the TB case detection rate

## Methods and materials

This study protocol has been reported in accordance with CONSORT statement: extension to cluster randomized trials standard protocol guidelines [29].

### Study design and participants

A parallel group cluster randomized controlled trial with two arms (i.e., intervention and control arms) will be conducted in six districts of the South Gondar Zone. The outcome of interest will be case detection rate, diagnosis delay, and catastrophic costs. The control arm will include the routine standard TB cares. In the intervention arm, TB-suspected individuals (i.e., people with coughs for more than 2 weeks, fever, chills, night sweats, anorexia, weight loss, chest pain, hemoptysis, lymphadenopathy, fatigue, or growth failure in children) will be identified by the traditional healer in traditional care centers (traditional healer clinics, holy water, mosques) and referred to modern health care centers (health facilities) where there is TB diagnostic service.

### Study settings

The study will be conducted in the South Gondar Zone, Amhara regional state, northwest Ethiopia. South Gondar is one of the zones found in the Amhara region. The South Gondar Zone is bounded by East Gojam to the south, West Gojam and Bahir Dar to the southwest, Lake Tina to the west, North Gondar to the north, Wag Hemra to the northeast, North Wollo to the east, and South Wollo to the southeast. The zone covers over 14,095 km^2^ roughly. The zone has 13 rural districts, 8 town administrations (two of them have health offices), and 404 kebeles which are the lowest administrative units in the districts. According to the 2007 Ethiopia Census, the zone had a total population of 2,051,738, of whom 1,041,061 are men and 1,010,677 were women [30]. According to the zonal population projection estimate, the total population increased from 2.05 million in 2007 to approximately 2.6 million in 2020. There are more than 10 hospitals, 96 health centers, and 397 health posts. TB treatment and preventive measures are taking place in all health facilities. The direct observation therapy (DOTS) program is applied in all government health facilities.

### Interventions

The intervention comprised three key components, which include referral linkage from traditional to modern health care, training of health professionals and traditional care providers in three different rounds to increase their knowledge, attitude, and skills toward the referral systems, as well as TB screening at traditional health care sites. TB screening and diagnosis services will be done collaboratively between traditional healthcare providers and modern healthcare providers. This study defined traditional care providers as practitioners that provide informal patient care, which includes traditional healers and religious leaders. A referral linkage model will be used to detect TB cases in traditional care sites. What we add to the usual practice is screening TB-suspected patients from traditional care centers and referring them to health facilities to improve TB case detection, reduce the costs of TB, and avoid diagnosis delays. The interventions will have the following four phases.

#### Phase 1: Preliminary phase or training material preparation phase

An intervention package, including training materials, will be developed in the preliminary phases of the intervention. Experts will be invited to develop training manuals. Physicians, public health experts, and language professionals will be invited to comment on the contents, depth, readability, and understandability of the training materials. To further enrich the training manual, a workshop will be conducted, and invited experts will give their opinions. The training material will be comprehensive and comprise a domain that includes knowledge, attitude, and skill. It will contain detailed information about TB including the causes, signs, and symptoms, mode of transmission, screening and diagnosis approach, case detection methods, ways to improve treatment outcome, the benefit of early detection and treatment, complications of late diagnosis, and control and prevention mechanisms of TB. The training will also include models used to integrate traditional and modern healthcare systems. The training manual will be approved by senior experts.

#### Phase 2: Training provision

Training will be given to both traditional and modern care practitioners in three different rounds. In the first round, traditional practitioners (traditional healers and religious leaders) will be trained for 5 days. Two days of training will be given to healthcare providers (TB focal persons) in the second round, and 1-day training will be carried out in the third round at the end of the sixth month after the first round of training. The training will be given by researchers and a person having training of trainers [4] certificates at the regional health bureau. Participants that can score 75% on the post-test in the knowledge and attitude domains and are skilled in practice will be considered part of the intervention team. A pilot test will be conducted outside the study area on 5% of the project sample size based on Connelly suggestion [31]. Based on the pilot findings and experts’ judgment, the intervention package will be refined. Before the actual intervention implementation, baseline data will be collected and analyzed.

#### Phase 3: Screening and referral of TB-suspected cases

In this phase, a full-blown intervention will be implemented. Traditional healers will screen their clients for TB using standardized screening tools. All suspected TB cases at traditional healer centers, holy water, and mosques will be referred to the nearby health facilities in the intervention districts. A trained TB focal person will screen and diagnose suspected cases based on the national TB treatment guidelines. The content of the interventions, duration, frequency, and dosages are presented in table 1 in Appendix 1.

The progress and evaluation of the intervention will be assessed quarterly and at 6 months. Regular monthly supervision of the implementation will be done by assigned experts. Intermediate outcomes will be assessed during the third and sixth months of the intervention by trained assessors.

#### Phase 4: The end-line evaluation

End-line outcome assessment will be done by trained assessors. The comparison will be done between the end-line and baseline outcomes (case detection rate, cost of TB, and diagnosis delay) between the intervention and control groups.

### Randomization and recruitment

Randomization will be done at the district level. There are thirteen districts and two town administrations. Among a total of districts and town administrations, six districts and/or town administrations in South Gondar Zone will be selected using the random.org software by considering the rule of thumb assumption that covers 40% of districts and town administrations. Thus, the selected districts/town administrations will be assigned to the intervention and control arms with a 1:1 allocation ratio. This means three districts or town administrations will be randomly assigned to the intervention arm, and the other three districts or town administrations will be assigned to the control arm. District or town size will not be considered in the randomization process. All health facilities in the intervention and control arms fulfilling the eligibility criteria will be included in the study. The eligibility criteria for the inclusion and/or exclusion of health facilities will be based on the availability of TB diagnostic and treatment services, including the microscope diagnostic service and DOT programs. Participants will recruit to the study when confirmed they have any form of TB and are registered to the DOT programs. Participants will enter into the trial individually based, and patients will recruit continuously until the desired sample size achieve. Recruitment of participants will be from July 1 to September 30, 2022. The statistician who allocated the intervention and control arms, an expert who assesses the outcomes, participants who will be assigned to intervention and control arms, and healthcare providers who provide TB care will not be aware of who were assigned to the intervention and control arms. The project manager and/or investigators will be unblinded.

### Data safety and monitoring

Data monitoring and interim analyses will be done by the data safety and monitoring committee. The trial data safety and adverse effect monitoring committee (DSMC) will be established to monitor the adverse effects of the intervention on patients. The committee will be formed from TB expertise, academia, stakeholders, planners, and officials. Adverse risks or harms will not be possible because the intervention will not directly involve any invasive procedures or will not administer drugs that affect the study participants. The DSMC will be used as the protocol for data reporting and interim analyses that minimize bias. Then, the committee will give recommendations based on their findings, either continuing the trial as planned or stopping early for hazards, or stopping the intervention because continuing the intervention is futile for the patient and community [32–35]. The committee will meet once a month and give feedback based on their assessment findings. The committee will recommend discontinuing the intervention when the intervention brings a negative impact on the health, social, cultural, spiritual, privacy, and violation of patient confidentiality. An interim analysis will be used to evaluate the data from an ongoing trial and address the primary research question and modify the intervention. A trial steering committee (TSC) will be formed from Bahir Dar University College of Medicine and health sciences ethics review board members and post-graduate office. The responsibilities of the TSC will be used to approve the main study protocol and any amendments, monitor and supervise the trial toward its interim analysis, and resolve problems brought by the trial coordinating team.

### Trial adherence and retention strategies

Adherence of the participants in both the intervention and control groups will be assessed using participants’ self-reports and direct observation by trained field supervisors. Regular communication and feedback will be made between the supervisors and the traditional care providers and healthcare workers. The project manager will be regularly communicated with supervisors, traditional care providers, and TB focal persons to minimize the attrition rate of participants in the intervention and control arms. Those participants who discontinue and completed the follow-up from the intervention and the number of outcomes that occurred during follow-up will be reported in the final dissertation.

### Patient public involvement

During the development of this research project, formative consultation occurred with the community advisory group and stakeholders including, Amhara Public Health Institute, local TB control program leaders, TB patients, traditional healers, and faith leaders through face-to-face interviews and focus group discussions, as part of our previous qualitative study [36]. The community and consumer representatives will be involved at each stage of the research project, including the planning, implementation, follow-up, and dissemination of research findings.

### Control groups

The control sites will follow the existing passive case-finding system (self-referral patients to nearby health facilities that use the same national guidelines to treat TB). The findings obtained from the control groups will be compared with those obtained from the intervention groups. Finally, changes between the two groups will be assessed and concluded.

### Trial status

Unique Protocol ID: 353/2021.

ClinicalTrials.gov ID: NCT05236452.

The date recruitment began: July 1, 2022.

Registration date: July 22, 2022.

Approximate date when recruitment will be completed: December 30, 2023 [anticipated].

### Outcomes of interest

The study will have three outcomes of interest. The primary outcome is the TB case detection rate. The secondary outcomes of interests are diagnosis delay and costs of TB care. The case detection rate will be expressed as percentage. The diagnosis delay will be defined as the time interval between the onset of symptoms and the confirmation of TB diagnosis, which includes patient delay and health system delay. The cost of TB care will include both direct and indirect costs of the patient for TB care and treatment as collected from the patients themselves and their medical records. Direct costs include out-of-pocket payments for medical services (counseling, drugs, laboratory tests, X-rays, and hospital stays) and non-medical services such as transportation, food, and accommodation costs.

### Definition of terms

Important variables are defined according to the researchers’ operation used in the research and all definitions are annexed in Appendix 2.

### Sample size determination

#### Sample size for TB diagnosis delay among tuberculosis patients

To estimate the effect of integrating traditional care with modern care on diagnosis delay, the sample size will be calculated using the two-sample comparison of proportions formula in STATA version 16. A chi-squared test with two independent samples will be used to calculate the sample size using diagnosis delay. By considering the previous study conducted in the Amhara region with the proportion of diagnosis delay P1 = 59.9% [24] and by assuming that the effect of the intervention on diagnosis delay decreased by 14% in the intervention group as compared to the control, P2 = 45.9%. By considering the probability of a type 1 error of 0.05, 95% CI, power of 80%, and a 10% non-response rate, the total sample size will be 438. Since the study is a multicenter, individually randomized trial, the design effect will be used. According to the multicenter design effect formula, the design effect formula, the $\mathrm{design\ effect}=\frac{\upsigma {e}^{2}}{\sigma {e}^{2}+\sigma {B}^{2}}$ = 1 − *ρ*. The majority of formulas applied to multicenter designs have a better relative accuracy (ICC less than 0.052) for designs including more than 10 centers. Assuming that randomized will be balanced and stratified on centers that have equal group sizes [37], assuming the variation between the centers will be 0.05, the design effect = 1 – *ρ* = 0.95. Therefore, the final sample size is calculated as sample size * design effect; 438 * 0.95 = 416 (208 for each arm).

#### Sample size calculation for cost of TB care among tuberculosis patients

To estimate the effect of integrating traditional care with modern care on the cost of TB care will be considered. The sample size will be calculated by using the two-sample comparison of proportions formula using STATA version 16. A chi-squared of the two independent samples will be used to calculate the sample size using household catastrophic cost incurred that catastrophic household expenditure of P1 = 40% [38] and assuming that the effect of the intervention on the cost of TB care decreased by 13% in the intervention group as compared to the control, P2 = 27% and using, 95% CI, power of 80%, and a type I error of 0.05, considering 10% non-response rate, the final sample size was 453. Since the study is a multicenter individually randomized trial, design effect will be used. According to the multicenter design effect formula, design effect formula is $\mathrm{design\ effect}=\frac{\upsigma {e}^{2}}{\sigma {e}^{2}+\sigma {B}^{2}}$ = 1 − *ρ*. The majority of formulas applied on multicenter design with better accuracy relative difference (ICC lesser than 0.052) for designs including more than 10 centers. Assuming that randomization will be balanced and stratified based on centers that have equal group sizes [37]. Assuming the variation between the centers is 0.05, design effect = 1 – *ρ* = 0.95. Therefore, the final sample size will be calculated as sample size * design effect 453 * 0.95 = 428 (214 in the intervention arm and 214 in the control arm).

#### Sample size calculation for TB detection rate

Since the case detection rate will be calculated by considering the WHO estimator for the year 2023 per 100,000 populations. Estimating the number of TB cases for that year will be computed by using total cases in the year/100,100 * total population found in the catchment area multiplied by 100, expressed as a percentage. Therefore, all cases that are confirmed as TB patients in the selected districts will be considered as the final sample size.

The number of clusters involved in the study was determined using the following formula [39]: The number of clusters (*K*) required in each arm for unequal cluster sizes was also determined using the formula [40]: *K* = *n* [1 + ((CoV2 + 1) *m* − 1) ICC]/*m*′], where Cov2 = coefficient of variation of 0.25, effective ICC value of 0.03 for cluster-level ICC value of TB detection rate, *n* = sample size under individual randomization, *m* = average number of individuals found in each cluster was 18. From 40 clusters, with unequal clusters for intervention and control arms, 24 clusters included in the intervention, and 16 clusters in the control arm.

### Data collection

Data will be collected on the dependent variables (i.e., case detection, diagnosis delay, and cost of TB care) and independent variables (i.e., socio-demographic, health-seeking behavior variables, patient knowledge of TB, healthcare accessibility, distance, contact history, and support). Secondary data will be collected from the patient’s medical records, and primary data will be collected using a structured questionnaire adapted from previous literature. The structured questionnaire is composed of socio-demographic and economic factors, health-seeking behaviors, patient knowledge, service accessibility, stigma, contact history, the catastrophic cost of TB, diagnosis delay, and healthcare initiation. The questionnaire will be translated into the national language (Amharic) spoken by almost all residents in the study area. The data will be collected using face-to-face interview questionnaires. The data will be collected by BSc nurses or public health officers who had data collection experience. Nurses or public health officers who had MSc/MPH degrees will be selected for data collector supervisors. The data will be collected at baseline and at the end of 1 year of the intervention.

### Measurements

The case detection rate will be calculated as the number of cases notified divided by the number of cases estimated for that year, expressed as a percentage [41]. The WHO estimator for the year 2022 was 132 per 100,000. Estimating the number of TB cases for that year will be computed by using that year/100,100 * total population of the catchment area. Estimating the number of TB cases for that year will be computed by using total cases in the year/100,100 * total population found in the catchment area multiplied by 100, expressed as a percentage.

Patient costs and their annual incomes collected in Ethiopian birr will be converted to US dollars ($) with an average exchange rate for the dates during which data collection took place. The total costs will be considered the sum of the entire direct and indirect costs of TB illness to patients. Out-of-pocket payment for health care (medical) will be the direct payment made for medical care directly by individuals at the time of service using minus insurance reimbursement. Out-of-pocket costs will be a payment for formal medical services, informal traditional practitioners, clinics, health centers, pharmacies, and hospitals for medical services and products such as diagnosis, treatment, and medicine. Out-of-pocket payment net will be a total out-of-pocket payment (medical and non-medical) minus any reimbursement received for payments made. The direct costs will be calculated by the sum of any payment affected by medical and non-medical costs. The medical costs will be the sum of out-of-pocket payments for TB diagnosis and treatment made by TB patients in a given household buying medicines, payments for diagnostic tests, and net reimbursements. The direct non-medical costs were out-of-pocket payments for transport, accommodation, and food before and during TB diagnosis. An indirect cost is costs incurred as a result of TB healthcare seeking and hospitalization, during the TB episode. The total period of absence (in hours) will be multiplied by the hourly wage rate of the absent worker.

Catastrophic costs due to TB: total costs (indirect and direct combined) exceeding a given threshold of the household’s annual income, here defined as exceeding 20%, as recommended by WHO [42].

### Data quality assurance

Data collectors will be recruited based on their experience with a minimum educational qualification of a BSc in nursing and public health officer. Also, supervisors will be recruited based on previous experience in data collection and supervision and a minimum qualification of an MSc or MPH degree. Training will be given to data collectors and supervisors for 1 day about the aim, methodology, sampling technique, ethical issues, and data collection instrument and procedure. Data collection tools will be judged by professional and language experts. The appropriateness of the tool will be checked by conducting a pretest, and the necessary amendments will be made based on the pretest results. Then, baseline data will be collected to compare the pre-and post-intervention findings of both intervention and control arms. The researcher will regularly communicate with the data collectors and supervisors, with face-to-face scheduled meetings and virtual meetings to discuss any issues that arise during the data collection. To minimize data entry errors, a double data entry method will be used. To avoid information contamination, the buffering zone will leave between the intervention and control clusters. Outcome measurements will be performed in the same manner in both arms. Training will be provided for the outcome assessor and the data will be analyzed using intention-to-treat principles [43]. To ensure methodological quality, a Consolidated Standards of Reporting Trials 2010 statement extension to cluster randomized control trial study guidelines will be used [44]. In addition, to ensure better reporting of the intervention, the template for the Intervention Description and Replication (TIDieR) checklist will be used to increase methodological quality [45].

### Data processing and management

In the field, data will be checked for consistency and completeness every day by supervisors and the principal investigator. Then, a data entry template will be created on EpiData version 4.6 based on coded responses. Data will be entered by data clerks using EPiData software about 10% will be randomly double-checked by the principal investigator. The data will be then exported to STATA version 14 for further analysis. Data coding and cleaning will be done to check for inconsistencies, outliers, and missing values. Finally, the cleaned data will be processed and analyzed using STATA. Before further processing of the data, numeric data will be checked for normality using normality plots (Q-Q plots and/or histograms) or normality tests.

### Data analysis

Descriptive statistics will be used to summarize the baseline data. A chi-square test will be performed to compare the baseline characteristics of the intervention and control groups. Comparisons of detection rate, diagnosis delay, and costs of TB care between and within the intervention and control groups will be done using independent samples and paired sample *t*-tests, respectively. The independent *t*-test will be also used for the knowledge and attitude of post-test results of practitioners.

Logistic regression analysis will be used to determine the factors associated with case detection rate and diagnosis delay. A univariable logistic regression model will be fitted, and variables having a *p*-value less than 0.25 in the univariable logistic regression model will be fitted into the multivariable models. Variables with a *p*-value < 0.05 in the multivariable logistic regression model can be considered statistically significant. Crude and adjusted odds ratio (OR) with 95% confidence interval (CI) will be calculated to measure the strength of association between the dependent and independent variables. The fitness of the logistic regression model will be checked using the Hosmer–Lemeshow goodness of fitness test at *p*-value > 0.05. Multicollinearity between explanatory variables will be checked by a variance inflation factor (VIF), and variables with a VIF > 5 will be excluded from the final models.

Generalized linear mixed models (GLMM) will be used to assess the impact of the intervention on the change of cost of TB care. This GLM enables us to accommodate the correlation of observations due to the pre and post-intervention measurements and clustering of individuals within randomly selected clusters. Model fitting will be checked before the analysis is done. The effects of potential confounding factors will be controlled. The variance of the cluster-level residual errors will be computed using the intercept-only model. The variation between and within the groups will be analyzed.

Subgroup analysis will be employed to explore whether there is evidence that the detection variation depends on certain patients or facility characteristics. Covariate adjusted analysis will also employ to refine the analysis of case detection variation in the intervention and control arms the fact that some baseline characteristics may be related to outcome and unbalanced between the groups.

### Dissemination plan

We plan to disseminate the results of the study through publication in peer-reviewed international journals, presentation at national and international conferences, and submission to beneficiaries, including governmental and non-governmental organizations. In addition, we will disseminate the results through reports to Bahir Dar University, press conferences, and website displays.

### Project timeline

The timeline of enrollment, interventions, and assessments of the project is well set. The enrollment started in July 2022, and the project will be closed in January 2024. The detailed schedule is annexed in Appendix 3.

## Discussion

This cluster-randomized controlled trial will test the effectiveness of a strategy that integrates traditional healthcare into the modern healthcare system for the control and prevention of TB in northwest Ethiopia, where nearly 90% of the population seeks care from traditional care systems. Although community TB screening has been conducted by community health extension workers, traditional care centers and spiritual healing centers are not easily accessible for community screening. Thus, the integration of traditional care and modern care will be an effective intervention for improving the prevention and control of TB programs. Our study adds screening suspected patients at the place where people gather together at traditional care and holy water centers for healing. Since more than one-third of TB cases are undiagnosed in our community [13], implementing screening for suspected cases in the holy water and traditional care centers probably improves the number of missed cases in the community. In addition, TB patients are diagnosed lately after the onset of the disease and excess expenditure of household costs. This trial will provide information on the effectiveness of traditional and modern health care integration to improve TB case detection and early diagnosis and treatment as well as to reduce the catastrophic cost of TB.

This study may be difficult to reach people who are referred to a health facility by a traditional healer or religious leader for suspected TB, but who do not have a telephone and cannot go to the health facility to which they were referred for various reasons. The other limitation is the project manager and/or investigators will not be blinded.

## Sponsor

The sponsor of this study is Bahir Dar University College of Medicine and Health Sciences. The sponsor has not played a role in the study design; collection, management, analysis, and interpretation of the data; writing of the report; and the decision to submit the report for publication.

### Supplementary Information

**Additional file 1.**

## Figures and Tables

**Table 2:**
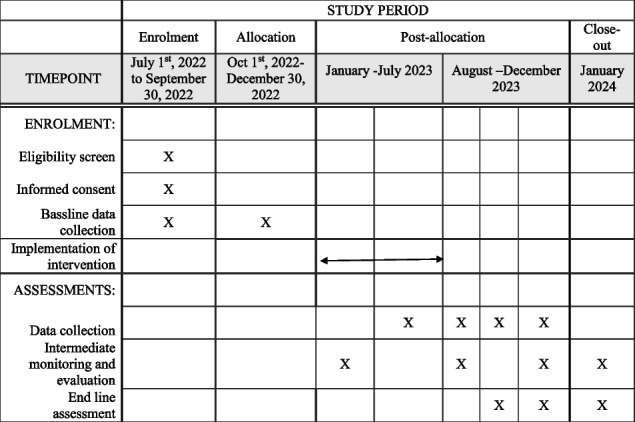
Timeline of recommended content for the schedule of enrolment, interventions, and assessments. The enrollment started in July 2022, and the project will be closed in January 2024

## Data Availability

Data are available from the corresponding author and are disclosed as per request by the journal.

## Appendix 1

**Table 1:** Intervetion packages; methods, frequency, duration, dosage and components of the intervention

Activities	Methods	Frequency, duration and doses	Components
Training for both categories of practitioners (traditional and modern care providers)	Group-based training will be provided by researchers	• The training will be given three times • 1st round for 5 days • 2nd round for 1 day at 3rd months • 3rd round for 1 day at 6th months	• Definition and overview of TB • Causes/etiology • Sign and symptoms • Transmission • Diagnosis • Treatment • Complications • Prevention and control • Advantages of early TB diagnosis and treatment • TB screening and detection
TB screening	• Collect both subjective and objective data • Role-play sessions will be performed	Every case every day for 1 year	• Take the history (any history of previous TB infection and any contact history • Check any signs and symptoms of TB cases
Screening patients who need spiritual support by healthcare providers	Group-based training and demonstration and role-play of how healthcare providers screen patients who need spiritual support	Every case every day for 1 year	Use any signs and symptoms of stress, such as sadness, frustration, or emotional instability, as an indicator
Referral linkage	Group-based training and individual-based practical/demonstration and role-play sessions will be conducted on how to refer suspected patients	Every case every day for 1 year • Sample referral • Patient referral	• Patient identification • At least one sign and symptoms of TB infection

## Appendix 2

### Definition of terms

Acceptability: Acceptability refers to the agreement of practitioners and other stakeholders on the appropriateness, suitability, adequacy, or effectiveness of the intervention.

Active case findings: Active case finding intervention consisting of community-based approaches (TB screening outreaches, community outreach/house-to-house screening, and contact tracing of members of households of TB patients) and systematic screening of target groups (pregnant women, women attending maternal and child health clinics, outpatient department, and HIV-infected individuals).

Catastrophic costs: Total costs (indirect and direct combined) incurred during illness and treatment exceeding a given threshold (20%) of the household’s annual income. Total direct and indirect costs incurred from the onset of symptoms to the end of TB treatment will be considered the numerator, as well as annual household income, will be considered the denominator [46].

Case detection: Case detection is defined as TB being diagnosed in a patient and is reported within the national surveillance system and then to WHO.

Case detection rate: The case detection rate is calculated as the number of cases notified divided by the number of cases estimated for that year, expressed as a percentage [41].

Case notification: Notification is the process of reporting diagnosed TB cases to WHO; the data collected by this process.

Diagnostic delay: Time interval between the onset of symptoms and labeling of the patient as a patient with TB (TB diagnosis).

Feasibility: An umbrella term used to describe any type of study relating to the preparation for the main study. A feasibility study is an analysis that takes all of a project’s relevant factors into account including economic, technical, legal, and scheduling considerations to ascertain the likelihood of completing the project successfully [47].

Health care system delay: Health system delay is the time interval between the date of health-seeking behavior at a health care provider and the initiation of anti-TB treatment.

Holy water: Holy water is water that has been blessed by a member of the clergy and used in baptism to heal individuals of their physical, psychological, and spiritual illnesses.

Patient delay: Patient delay is the time interval between the onset of symptoms and presentation to a health care provider.

TB suspect: Any person who presents with symptoms or signs suggestive of TB. The most common symptom of pulmonary TB is a productive cough lasting more than 2 weeks, which may be accompanied by other respiratory symptoms (shortness of breath, chest pains, hemoptysis), and/or constitutional symptoms (loss of appetite, weight loss, fever, night sweats, and fatigue) [48].

Traditional healer: The term traditional healer refers to those practitioners who provide any health and health-related healing services using their indigenous knowledge, and who are registered with legitimate authorities, while religious healers refer to religious leaders who provide healing services at holy water sites [49].

Traditional medicine: TM is the total of the knowledge, skills, and practices based on the theories, beliefs, and experiences indigenous to different cultures, whether explicable or not, used in the maintenance of health as well as the prevention, diagnosis, improvement, or treatment of physical and mental illness [27].

Total delay: The total delay is the time interval from the onset of illness until the initiation of anti-TB drugs. It is the sum of two-time intervals: the diagnostic delay and the treatment delay. The total delay is also the sum of patient and healthcare system delays.

Treatment delay: Time interval between the TB diagnosis and the initiation of anti-TB drugs.

## Appendix 4

### Consent to take part in research

I ……………………………………… voluntarily agree to participate in this research study. I understand that even if I agree to participate now, I can withdraw at any time or refuse to answer any question without any consequences of any kind. I understand that I can withdraw permission to use data from my interview within 2 weeks after the interview, in which case the material will be deleted. I have had the purpose and nature of the study explained to me in writing, and I have had the opportunity to ask questions about the study. I understand that I will not benefit directly from participating in this research. I agree to my interview being audio-recorded. I understand that all information I provide for this study will be treated confidentially. I understand that in any report on the results of this research, my identity will remain anonymous. This will be done by changing my name and disguising any details of my interview which may reveal my identity or the identity of the people I speak about. I understand that disguised extracts from my interview may be quoted in the study. I understand that if I inform the researcher that I or someone else is at risk of harm they may have to report this to the relevant authorities, and they will discuss this with me first but may be required to report with or without my permission. I understand that signed consent forms and original audio recordings will be retained in a locked computer with the primary investigator or locked cabinet. I understand that under freedom of information legalization, I am entitled to access the information I have provided at any time while it is in storage as specified above. I understand that I am free to contact any of the people involved in the research to seek further clarification and information.

By Desalegne Amare (MSC, PhD candidates).

Bahir Dar University College of Medicine and health sciences.

Email: desalegnezelellw@gmail.com.

Mobil: + 251929466624.

Signature of interview: ____________________; Date: ________________________.

Signature of participant: ___________________; Date: ________________________.

I believe the participant is giving informed consent to participate in this study.

Signature of researcher: ____________________; Date: _______________________.

## Appendix 5

### Assent form

My name is ______________. The purpose of the study will be to assess the effect of integrating traditional tuberculosis care with modern health care on case detection, diagnosis delay, and costs of TB care. We are randomly selecting you to participate in this research. Your participation is important to address the research objective stated above.

One of your parents or guardians has already given their permission for you to be part of this study, and now it is your turn to decide. If you decide that you want to be part of this study, you will be asked to participate in this study. Participating in the study has not any hurt on you but you will be asked few information about your experience in TB diagnosis and treatment. When we are finished with this study, we will write a report about what was learned. This report will not include your name or that you were in the study. You do not have to participate in this study if you do not want to. If you have questions or decide to stop after we begin, that is okay; just tell your parents and withdraw at any time. If you want to be in this study, please write your name and sign below.

Check one:

_________ I want to be in the study.

_________ I do NOT want to be in the study.

Your name: _____________________________.

Your signature: __________________________.

Person to contact:

Principal Investigator: Desalegne Amare.

Bahir Dar University.

Mob: + 251929466624.

E-mail: desalegnezelellw@gmail.com.
